# Editorial: Coronavirus disease (COVID-19): mechanistic basic, translational, and clinical research aimed at identification of effective preventive and therapeutic strategies

**DOI:** 10.3389/fphar.2023.1247765

**Published:** 2023-07-13

**Authors:** Paolo Montuschi, Fatih M. Uckun, Alastair George Stewart, Paola Patrignani

**Affiliations:** ^1^ Faculty of Medicine, Imperial College London, National Heart and Lung Institute, London, United Kingdom; ^2^ Pharmacology, Faculty of Medicine, Catholic University of the Sacred Heart, Roma, Italy; ^3^ Ares Pharmaceuticals, LLC, St. Paul, MN, United States; ^4^ Department of Pharmacology and Therapeutics, University of Melbourne, Parkville, VIC, Australia; ^5^ Department of Neuroscience, Imaging and Clinical Sciences and CAST, “G. d’Annunzio” University, Chieti, Italy

**Keywords:** coronavirus disease (COVID-19), inflammation, cytokine release syndrome, treatments of COVID-19, preventive strategies in COVID-19

The coronavirus disease 2019 (COVID-19) is a significant global health concern affecting society and the economy. Although our understanding of the risks associated with COVID-19 has improved, there are still medical challenges to overcome, and effective treatments for severe disease are not yet available. This Research Topic aimed to showcase recent discoveries and ongoing research efforts to help speed up recovery from COVID-19 and prevent life-threatening complications. It comprises four papers.

Based on the experience of the Health Center Grude (Bosnia and Herzegovina) in treating COVID-19 patients Vukoja et al. reported on work scheme, therapeutic approach, and management techniques, which have proven beneficial. They presented a case series showing how even severely ill patients can be treated entirely in primary care settings. The management is based on the timely and rational use of dexamethasone and various other pharmacological and nonpharmacological treatments. This approach might be particularly useful when tertiary care centers and academic institutions are overwhelmed.

The systemic inflammatory response in critical patients with COVID-19 is associated with alterations in blood lipid profiles. A significant reduction in the serum levels of apolipoprotein-A-I (ApoA-I), i.e., the major protein component of high-density lipoprotein (HDL), has been detected. This protein has many important functions, such as reducing systemic and lung inflammation, regulating the immune system, and preventing endothelial dysfunction and blood coagulation. In four immunocompromised patients with severe COVID-19 with cytokine release syndrome (CRS), also known as “cytokine storm,” that progressed despite standard-of-care therapy, Faguer et al. provided initial safety assessment and proof of concept for repeated infusions of CER-001, an ApoA-I-containing HDL mimetic, which was well-tolerated with no serious adverse events. These preliminary results are promising, but randomized controlled trials are required to assess the therapeutic potential of ApoA-I-containing HDL mimetics in these patients.

Using network pharmacology (NP), Jiang et al. explored potential endotypes and specific targets common to COVID-19 and rheumatoid arthritis (RA), ankylosing spondylitis (AS), and gouty arthritis (GA). They sought to elucidate the mechanism of action of Cepharanthine (CEP) as a potential treatment for COVID-19. CEP, a monomer component of traditional Chinese medicine primarily used to treat leukopenia, has shown promising therapeutic effects on COVID-19, but its specific molecular mechanism remains unclear. This study showed common potential targets, including tumor necrosis factor (TNF), interleukin (IL)-6 and IL-1β, and signaling pathways of IL-17 and TNF, with potential implications for cross-disease biomarker discovery and targeted therapies. This study provided new insights regarding the clinical potential of CEP in treating COVID-19 and its molecular mode of action.

The paper by Remuzzi et al. reported the work of the COVID-19 Committee of the Lincei Academy, which has reviewed the scientific evidence supporting the efficacy and safety of existing and new drugs/biologics for the preventing and treating of COVID-19 and its complications. This report provides an overview of the available evidence on drugs and biologics recommended by health authorities and experts, pointing out drugs that are not recommended or lack sufficient evidence for or against their use. Additionally, they discussed the safety of drugs used to treat COVID-19 co-morbidities. Particular attention was paid to further understanding COVID-19 pathophysiology to expedite the development and repurposing of safe and effective treatments.

